# Multiple Keratoacanthomas Following Moderna Messenger RNA-1273 COVID-19 Vaccination Resolved With 5-Fluorouracil Treatment: Case Report

**DOI:** 10.2196/41739

**Published:** 2022-12-01

**Authors:** Fahad Ahmed, Nashwah Memon, Adel Haque

**Affiliations:** 1 Department of Dermatology Perelman School of Medicine University of Pennsylvania Philadelphia, PA United States; 2 Lake Erie College of Osteopathic Medicine Bradenton, FL United States; 3 Department of Dermatology Jefferson Health Northeast Philadelphia, PA United States

**Keywords:** keratoacanthoma, COVID-19 vaccine, COVID-19, vaccine, treatment, Moderna, messenger RNA, side effects, vaccination, case report, oncology, tumor

## Abstract

Cutaneous reactions have been commonly associated with the Moderna messenger RNA (mRNA) COVID-19 vaccine. Among the reported cutaneous side effects, there have not been any associations reported yet regarding keratoacanthoma development after COVID-19 mRNA vaccination. We report a novel case of an 86-year-old man who experienced an eruption of multiple keratoacanthomas 2 weeks after inoculation with the Moderna mRNA-1273 vaccine that resolved following treatment with intralesional 5-fluorouracil.

## Introduction

The Moderna messenger RNA (mRNA) COVID-19 vaccine was granted emergency use authorization within the United States in December 2020. As vaccination administration expands, a growing body of evidence becomes available, allowing for a greater understanding of a novel vaccine’s adverse effects. A recent registry-based study by McMahon et al [1] documented 414 unique cases of cutaneous reactions after COVID-19 vaccination, with 343 (83%) occurring in patients who received the Moderna mRNA vaccine. In our study, we report a novel case of an eruption of multiple keratoacanthomas 2 weeks after inoculation with the Moderna mRNA-1273 vaccine that resolved following treatment with intralesional 5-fluorouracil.

## Case Report

**Figure 1:**
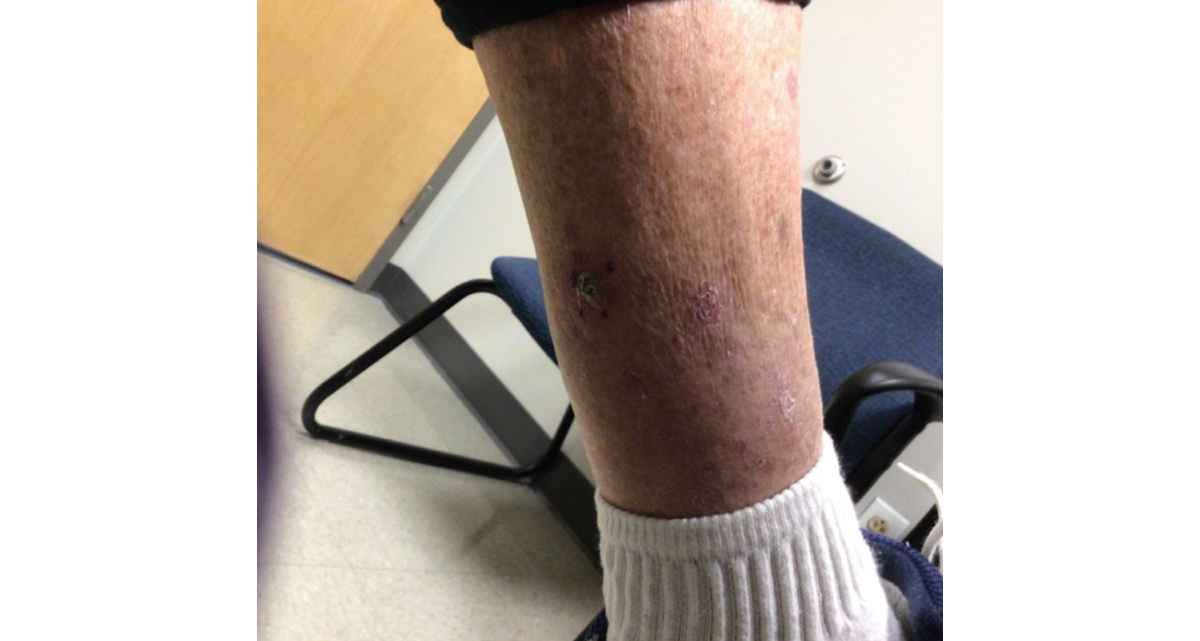
Clinical image taken on the day of the initial visit (March 2021).

**Figure 2:**
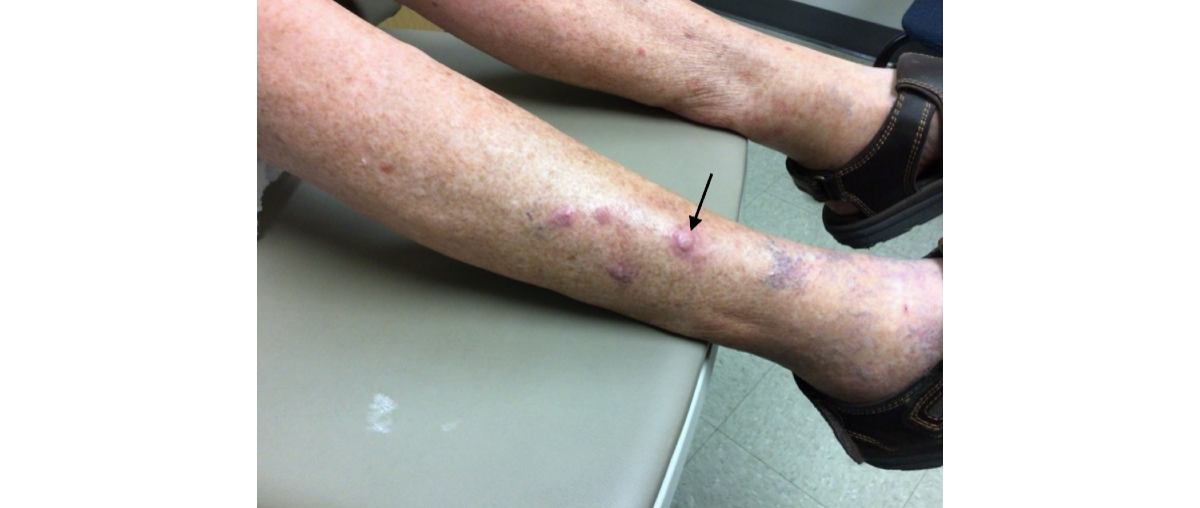
Clinical image taken at follow-up (August 2021). Arrow indicates biopsy site.

**Figure 3:**
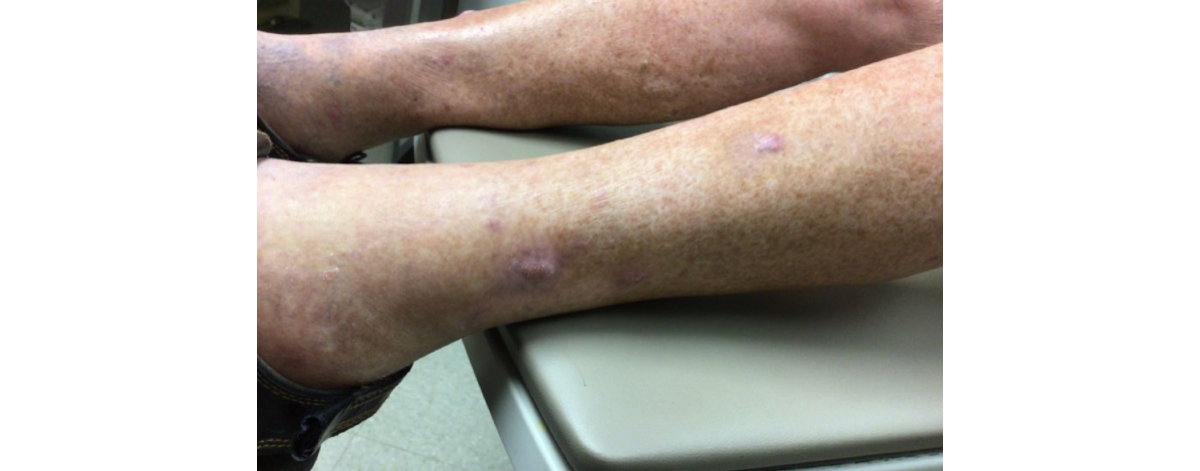
Clinical image taken at follow-up (August 2021).

**Figure 4:**
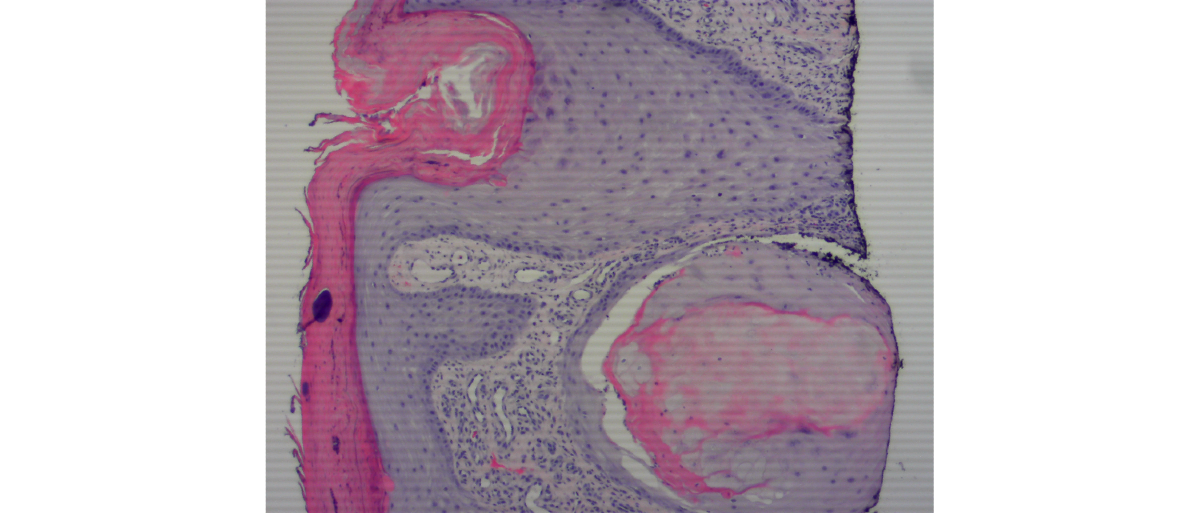
Histopathology of squamous cell carcinoma, keratoacanthoma type. This skin biopsy demonstrates atypical keratinocytes replacing the full thickness of normal epithelium. Cells with large hyperchromatic and atypical nuclei are seen at all levels. Atypical keratinocytes extend into the dermis as small islands. Scattered mitotic figures are also observed. A hematoxylin and eosin stain is used.

An 86-year-old man with no past medical history who had not recently initiated any medications received the second dose of the Moderna COVID-19 vaccine on March 2, 2021. On March 16, 2021, he began to experience severe pruritus of the lower pretibial area, and within 2 days, he noticed red nodules on the bilateral legs (7 on the right and 4 on the left; Figure 1). Prior to this date, the patient did not experience any previous cutaneous signs or symptoms that were concerning for keratoacanthomas on the bilateral legs. A shave biopsy of the left calf 1 week later demonstrated a well-differentiated keratoacanthoma-type squamous cell carcinoma (SCC). There were 11 other similar lesions at the time of initial presentation; however, none were biopsied. Of note, there were no lesions at the vaccination site. The lesion was cleared via Mohs surgery in 2 stages; however, the area was complicated by infection and required almost 2 months to heal. The remainder of the eruption was diagnosed as *prurigo nodularis*, and the patient was prescribed a clobetasol ointment, with minimal improvement. The patient then presented to our clinic in August 2021, at which point 2 scallop biopsies were taken from the right pretibial area (Figure 2 and Figure 3). The pathology results were consistent with SCC, keratoacanthoma type (Figure 4). Given the numerous lesions, the patient was treated with intralesional 5-fluorouracil with resolution over a 6-week period, that is, the patient was treated with 1.5 mL of 50-mg/mL 5-fluorouracil, which was injected intralesionally into each growth once per week for 6 weeks. Initially, the right leg was treated, requiring 3 weeks of treatment. Afterward, the left leg was treated, also requiring 3 weeks of treatment.

## Discussion

Keratoacanthomas represent tumors with atypical, highly differentiated squamous epithelia that typically arise on the head, neck, and extremities as volcano-like lesions that form quickly and have been shown to regress spontaneously. Keratoacanthomas share many histopathologic features with SCC, resulting in their recent reclassification as “squamous cell carcinoma, keratoacanthoma type.” Eruptive keratoacanthomas are a variant of keratoacanthomas that involve the appearance of multiple nodules in a short period. Although the etiology and pathophysiology of keratoacanthomas are widely considered to be multifactorial, immune status has been reported as a contributing risk factor. Similarly, our patient’s rapid keratoacanthoma development may have been influenced by this vaccine (ie, vaccination resulting in the development of a proinflammatory response). In one study, the tumor microenvironment and subsequent keratoacanthoma progression were shown to be influenced by the ratio of T helper 17 cells to regulatory T cells and proinflammatory and anti-inflammatory responses, respectively [2]. Multiple eruptive keratoacanthomas are also seen in some syndrome associations, including Muir-Torre syndrome, Ferguson-Smith disease, Grzybowski syndrome, *incontinentia pigmenti*, and xeroderma pigmentosum associations, and are also associated with human papillomavirus infection.

The Moderna COVID-19 vaccine is a relatively novel mRNA- and proinflammatory response–based technology. Although very rare, keratoacanthomas have been reported after pneumococcal and smallpox vaccine inoculation. Notably however, these vaccines do not use mRNA delivery technology [3,4]. Additionally, multiple cases of eruptive keratoacanthomas in short time frames following treatment with various immune modulators, such as leflunomide, pembrolizumab, and vemurafenib, have been reported in the literature [5-7]. Although the role of immunosuppression in the pathogenesis of SCC is well documented in the literature, the role of the immune system in the context of vaccine-induced keratoacanthomas or drug-induced keratoacanthomas is less well understood. In all stages of a keratoacanthoma (proliferative, maturation, and involution), the infiltration of lymphocytes has been demonstrated. These infiltrates could be responsible for the rapid tumor growth and tissue necrosis seen with keratoacanthomas [2]. Thus, an immune-mediated mechanism may be responsible for the dermatological adverse events resulting from vaccination with the mRNA-1273 COVID-19 vaccine.

Eruptive keratoacanthomas that do not show signs of regression can be a challenge to treat due to the number of lesions and the risks associated with surgical management in certain clinical settings (ie, patient age, comorbidities, and lesion severity). The efficacy of intralesional 5-fluorouracil—a chemotherapeutic agent—has not been studied extensively, although promising results have been reported in limited data sets. Kraus et al [8] reported that 96% (22/23) of the evaluable cutaneous SCCs in their study were completely cleared, as confirmed by histopathology. Adding to this evidence, Maxfield et al [9] recently demonstrated the resolution of 92% (158/172) of the cutaneous SCCs in their study with intralesional 5-fluorouracil, which is comparable in efficacy to Mohs surgery; 5-fluorouracil was injected at a concentration of 50 mg/mL, with volumes ranging from 0.2 to 2 mL per lesion, and in some cases, repeat injections were required at follow-up.

COVID-19 has shown a deadly predilection for individuals in the older population who become infected with SARS-CoV-2, with some studies showing case fatality rates and hospitalization rates as high as 14.8% and 18.4%, respectively [10]. The development of mRNA technology and the rapid production of the vaccines from Pfizer-BioNtech and Moderna have resulted in the reduction of mortality rates in the older population. Given these high mortality figures, all patients over the age of 65 years should be strongly encouraged to receive the vaccines; however, given their novelty, cutaneous eruptions, side effects, and associated treatments will need to be well recognized by dermatology providers. In the case of multiple eruptive keratoacanthomas in an older population with many comorbidities, 5-fluorouracil can be beneficial as a first-line, nonsurgical treatment option, especially for patients who are poor surgical candidates or areas that are difficult to heal or have a high risk for infection.

As our patient did not have any new or known risk factors for the development of eruptive keratoacanthomas on the bilateral legs, clinicians should be aware of Moderna COVID-19 vaccine–induced keratoacanthomas—a novel finding—as a potential occurrence following vaccination with the mRNA vaccine. As with any individual case report, we acknowledge the limitation of our report in determining the causation of eruptive keratoacanthomas following COVID-19 vaccination. However, our case will contribute to the limited clinical data on cutaneous, adverse COVID-19 vaccine side effects. Further reports and studies of any additional cases will be important for investigating the incidence and pathophysiology of this potential adverse reaction. Our patient’s 11 keratoacanthomas resolved after treatment with intralesional 5-fluorouracil, which can be considered as a first-line therapy for multiple keratoacanthomas in similar clinical contexts.

## Abbreviations

mRNA: messenger RNA
SCC: squamous cell carcinoma
